# Hypogravity simulation using the Variable Gravity Suspension System: A technical report

**DOI:** 10.1113/EP092172

**Published:** 2025-07-23

**Authors:** Patrick Swain, Anthony Swain, Filipa Santos, Luke Hughes, Kirsty Lindsay, Ishbel Lomax, Claire Bruce‐Martin, Nick Caplan

**Affiliations:** ^1^ Aerospace Medicine and Rehabilitation Laboratory, Department of Sport, Exercise, and Rehabilitation Northumbria University Newcastle upon Tyne UK; ^2^ Department of Physics University of Oxford Oxford UK; ^3^ Departamento de Física, Faculdade de Ciências Universidade de Lisboa Lisboa Portugal

**Keywords:** analogue, astronaut, bodyweight support, hypogravity, Mars, Moon

## Abstract

Human movement has evolved within Earth's gravitational environment (1 *g*; −9.81 m s^−2^). Future human exploration of terrestrial bodies, including the Moon (0.17 *g*; −1.62 m s^−2^) and Mars (0.38 *g*; −3.71 m s^−2^), will require astronauts to live and work within reduced gravitational environments (hypogravity). Progressing understanding of the physiological and biomechanical implications of movement in hypogravity will play a key role in supporting the expansion of humanity to terrestrial bodies beyond Earth, within our solar system. Ground‐based hypogravity analogues that enable the study of human movement are pivotal to developing knowledge in this field. Whole‐body suspension can serve as a resource‐efficient and accessible hypogravity analogue, yet only a limited number of such analogues exist globally. This technical report introduces a new hypogravity analogue facility: the Variable Gravity Suspension System (VGSS). The report introduces the VGSS and its theoretical framework, which enables simulation of both micro‐ and hypo‐gravity, presents proof‐of‐concept data regarding its ability to simulate hypogravity, and demonstrates the ability of the VGSS to facilitate locomotive and jumping activities in simulated hypogravity.

## Abstract (highlights)

- **What is the central question of this study?**
  This study introduces and demonstrates proof‐of‐concept for the Variable Gravity Suspension System (VGSS), a newly developed analogue for simulating hypogravity using head‐up tilt whole body suspension
- **What is the main finding and its importance?**
  Hypogravity between 0 and 0.2 *g* can be accurately simulated using the VGSS and allows users to perform movement in the sagittal plane, including locomotive and jumping activities.

## 1 INTRODUCTION

International space agencies are preparing to expand the human presence within the solar system beyond low‐Earth orbit (ISECG, 2018), with NASA's Artemis programme aiming to land humans on the south pole of the Moon (0.17 *g*; −1.62 m s^−2^), later followed by the first crewed mission to Mars (0.38 *g*; −3.71 m s^−2^) by the mid‐2030s (Creech et al., 2022; ISECG, 2018). With plans to develop the necessary technologies and infrastructure for sustaining human life on terrestrial bodies within our solar system for extended periods, it is vital that the effects of hypogravity (>0 *g* and <1 *g*) on human physiology and biomechanics are better understood.

Understanding the physiological and biomechanical consequences of human movement in hypogravity and when transitioning between micro‐ and hypo‐gravitational fields, such as during transit to and landing on the Moon/Mars and upon return to and landing on Earth, has useful applications to mission operations (De Martino et al., 2023). Life support system and countermeasure requirements, design of surface habitats, extra‐vehicular activity (EVA) suits and equipment/tools required for operational tasks, identification of risks to human health and performance, establishing fitness for duty standards, understanding requirements of EVAs (e.g., ambulation strategies/speeds, energy expenditure and fatigue) and crew training must be evidence‐based. Hypogravity research on humans is, however, rather limited. Richter and colleagues identified only 43 studies that have investigated the effects of hypogravity between 0.1 and 0.4 *g* on biomechanical or cardiopulmonary outcomes (Richter et al., 2017), and others have remarked that hypogravity research is fragmented and characterised by heterogeneous study contexts and experimental designs (De Martino et al., 2023; Lacquaniti et al., 2017; Sylos‐Labini et al., 2014).

The most ecologically valid setting from which to investigate the effects of lunar and Martian gravity on human movement would be the actual Moon and Mars, respectively; however, the challenges of accessibility have rendered any research of this kind completely impossible beyond the six Apollo Moon landings (1969–1972). When humans return to the Moon and eventually venture to Mars, it will still only be possible to study a limited number of astronauts per mission, a statistical challenge that has persisted throughout the era of human spaceflight. One of the most captivating sources of information about human movement in hypogravity to date is video footage from the Apollo Moon landings, in which astronauts can be seen preferentially selecting a hopping‐style gait to ambulate across the lunar surface and were susceptible to losing balance and falling, although one cannot dismiss the effects of the Apollo EVA spacesuit that had apparent restrictions on movement and visibility (Amovees, 2013; Scheuring et al., 2007).

Ground‐based analogues currently provide the most feasible means of conducting research into the physiological and biomechanical consequences of operating within a simulated hypogravity environment. Parabolic flight is the highest fidelity analogue as it allows passengers within the aircraft to experience ∼18–30 s of micro‐ or hypo‐gravity during each parabola (Shelhamer, 2016). The brief parabola period, however, only allows acute effects to be studied in a somewhat confined aircraft. Access to parabolic flights is also competitive, and the frequency of campaigns limits overall research opportunities. Head‐up tilt (HUT) bedrest, on the other hand, allows for the longitudinal examination of how physiological systems adapt to long‐term partial weight‐bearing (i.e., simulated hypogravity) exposure typically spanning days to months and how the body re‐adapts upon return to full weight‐bearing; however, movement is heavily restricted as the user is confined to a bed at a specific HUT angle (i.e., 9.5° (lunar gravity simulation) and 22.3° (Martian gravity simulation)) (Cavanagh et al., 2013). Readers are directed to NASA's ‘*Partial‐Gravity Analogs Workshop*’ technical memorandum for an overview of different cellular, animal and human space‐ and ground‐based hypogravity analogues (Barr et al., 2016).

Whole‐body suspension has been used for decades as a ground‐based analogue that allows for human movement in simulated hypogravity for minutes to several hours. A number of suspension models exist and operate in distinct ways (Sylos‐Labini et al., 2013, 2014). In brief, vertical suspension off‐loads a select portion of bodyweight by providing a controlled upwards force on the user through a body harness system (e.g., NASA's Active Response Gravity Offload System; ARGOS) (Bekdash et al., 2020). With an appropriate system design, vertical suspension can allow users to perform tasks within a three‐dimensional space under the effects of simulated hypogravity (e.g., material transport) that is particularly beneficial for astronaut surface EVA training. As only the centre of mass is unloaded, the limbs within the swing phase and the cardiovascular system still experience 1 *g* along the body's longitudinal axis that can impact research outcomes (Sylos‐Labini et al., 2013; Whittle et al., 2022).

Microgravity simulation can be achieved through horizontal suspension, where the orientation of the body with respect to the direction of Earth's gravitational field neutralises the component of gravity acting along the axial (head‐to‐foot) line of the body. The weight of individual body segments can be counterbalanced using vertical ropes that apply an upwards force equal to the respective body segment's weight, thus rendering that body segment weightless. By applying an axial force on the user through a subject‐loading or ‘gravity replacement’ device, different levels of partial weight‐bearing can be imparted onto the body (e.g., European Space Agency's Vertical Treadmill Facility; VTF) and can be used to reproduce the effects of how astronauts use the T2 treadmill on the International Space Station (Weber et al., 2019). Horizontal suspension combined with a loading device can also be viewed as a hypogravity analogue, as the user can experience 0–100% bodyweight. The application of an external axial force, typically through a shoulder and/or waist harness, does have some drawbacks, including said force unlikely to be uniformly experienced throughout the participant's body and possible constraints to user movement. The ability of the employed subject‐loading device to provide a constant axial force during activities must also be considered, as previous findings indicate that pull‐down force in users hopping in horizontal suspension up to 20 cm is non‐constant and variable between users and simulated hypogravity levels (Weber et al., 2019).

**FIGURE 1:**
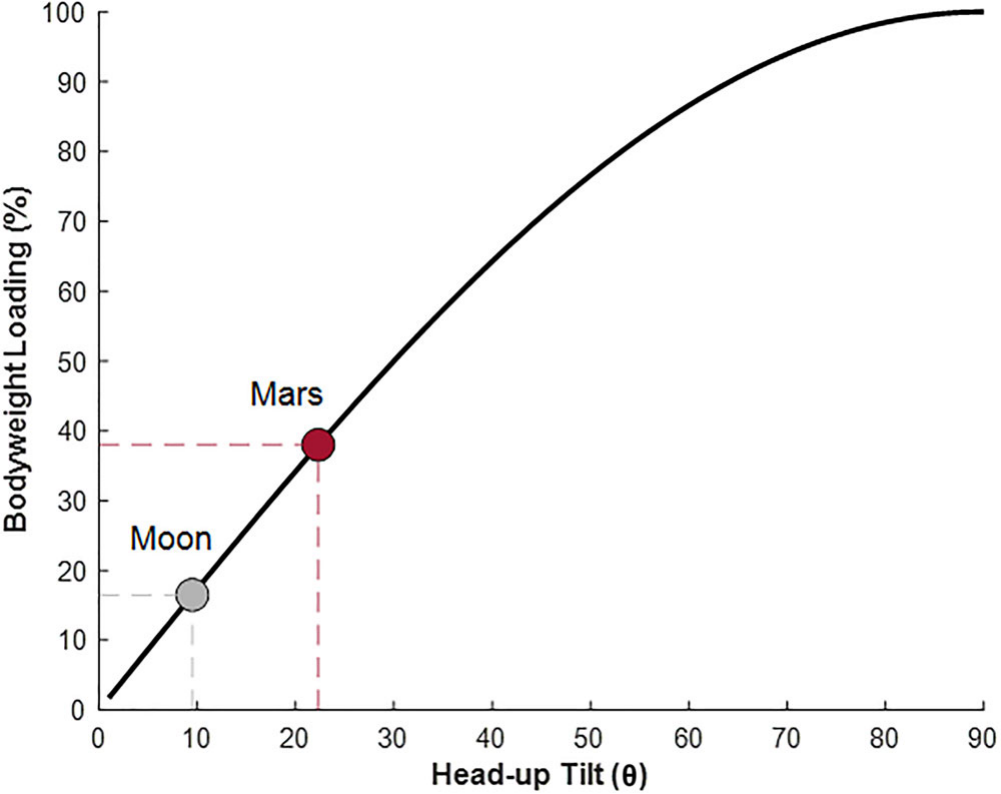
Relationship between user HUT angle (θ) from the horizontal plane and axial (head‐to‐toe) loading as a percentage of bodyweight in Earth's gravitational field (−9.81 m s^−2^). Markers for the Moon (●) and Mars (●) have been added at their corresponding HUT angles and bodyweight loads: 9.5° (∼17% bodyweight; 0.17 *g*) and 22.3° (∼38% bodyweight; 0.38 *g*), respectively. HUT, head‐up tilt.

HUT suspension orientates the body at a fixed HUT inclination relative to the horizontal plane resulting in a component of Earth's gravity (1 *g*) acting along the axial line of the body, thus generating the desired partial bodyweight (bodyweight = *mg* sin(θ), where *m* is the participant's mass and θ is the HUT angle) (Sylos‐Labini et al., 2014). Figure 1 illustrates the relationship between bodyweight loading and suspension angle. Suspension of the body at 9.5° and 22.3° HUT can, therefore, be used to mimic the gravitational acceleration of the Moon (0.17 *g*) and Mars (0.38 *g*), respectively.

An example of HUT suspension is the Reduced Gravity Walking Simulator developed by NASA in preparation for the Apollo Moon landings. Video footage of the system in operation can be found online, in which users can be seen walking, hopping, skipping, jumping and even performing a backflip, in simulated lunar gravity along a tilted walkway, resembling footage of the Apollo Moon walks (NASA, 1965). HUT suspension has the advantage that the participant experiences a hypogravitational acceleration along the same body axis as they would experience hypogravity on the Moon or Mars, with this same acceleration experienced in all body segments and without the constraints imposed by a harness for additional force application.

Few suspension‐based analogues exist globally, despite having the capability to be more accessible than other hypogravity analogues (e.g., parabolic flight) and being employed for research on a regular basis. Development of new HUT suspension facilities, therefore, can accelerate hypogravity‐related human movement research to support exploration missions (De Martino et al., 2023; Sylos‐Labini et al., 2014). Not only can hypogravity research have important applications to exploration programmes, but also terrestrial populations on Earth, a topic that has been discussed by several reviews that outline the various ways research in this field can benefit the development of novel technologies for gait rehabilitation, treatment strategies for accelerating recovery following injury/surgery, and promotion of movement in populations with neurological impairments (e.g., spinal cord injury, stroke and cerebral palsy) (De Martino et al., 2023; Lacquaniti et al., 2017; Sylos‐Labini et al., 2014).

The Variable Gravity Suspension System (VGSS) is a new micro‐/hypo‐gravity analogue using whole body suspension, developed by the Aerospace Medicine and Rehabilitation Laboratory in the UK. The aim of this technical report is to introduce the VGSS and its theoretical framework, demonstrate the validity and reproducibility of the system to simulate hypogravity at various HUT inclinations, and illustrate the feasibility of locomotory and jumping activities in simulated lunar gravity.

## 2 METHODS

### 2.1 System design

**FIGURE 2:**
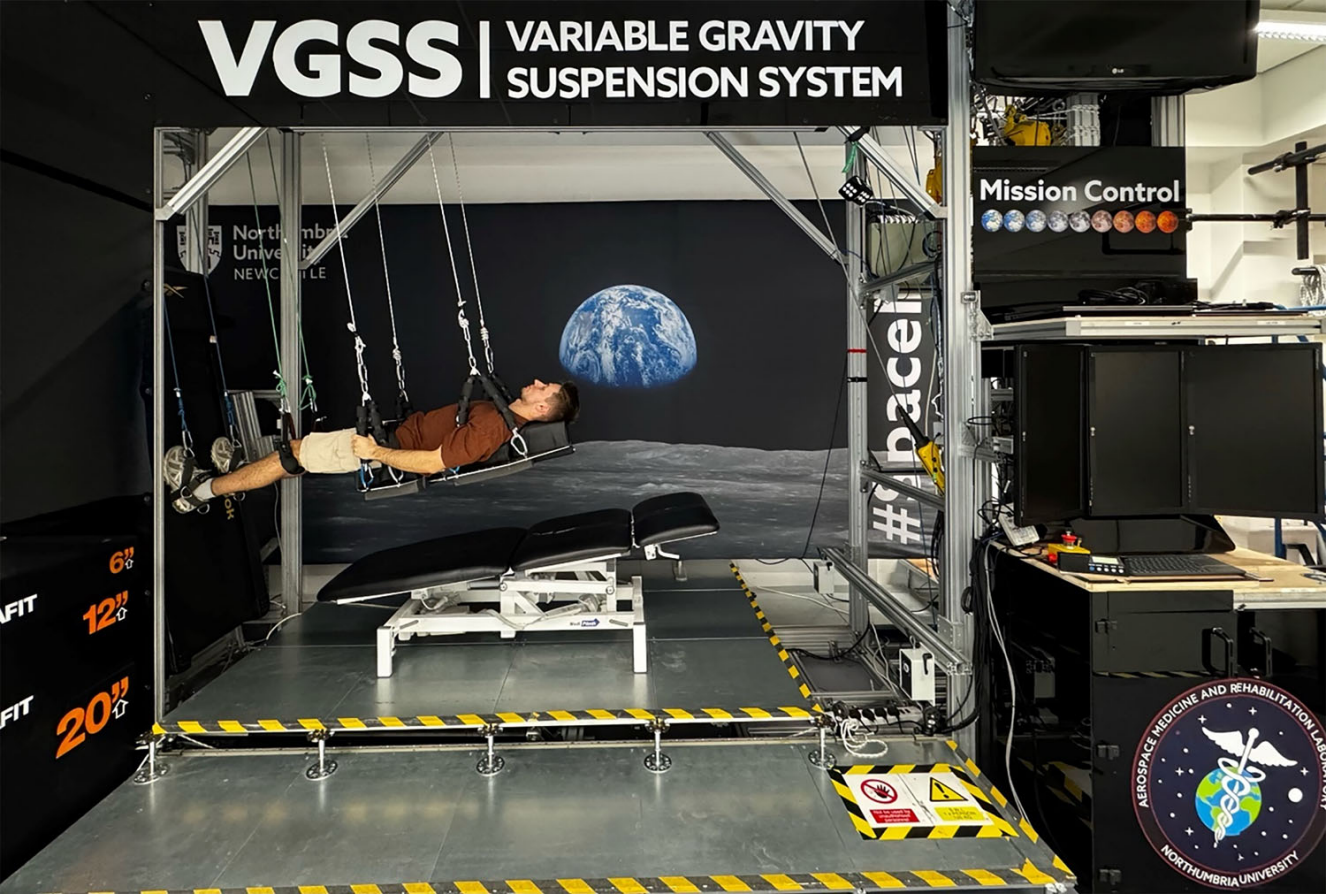
User standing in simulated lunar gravity (9.5° head‐up tilt) within the Variable Gravity Suspension System hypogravity analogue.

The VGSS frame is fabricated from aluminium strut profiles (45 mm × 45 mm or 45 mm × 90 mm Rexroth, Bosch, Lohr am Main, Germany). The main sections of the VGSS include (1) a user suspension and foot platform area, (2) an overhead rope and gantry system, and (3) spring‐balance housing (Figure 2). Users are suspended by six ropes in the central, open area of the VGSS. Each rope supports a specific body segment:

- Rope 1 (thorax): head, neck, thorax and arms
- Rope 2 (pelvis): pelvis
- Rope 3 (left knee): left thigh and part of the left lower leg
- Rope 4 (right knee): right thigh and part of the right lower leg
- Rope 5 (left ankle): part of the left lower leg and right foot
- Rope 6 (right ankle): part of the right lower legs and right foot

An electronic plinth (Ultrasound Couch, Medisave, Weymouth, UK) is positioned in the central open area of the VGSS for the participant to lie on during set‐up prior to suspension. The plinth can tilt and lift such that the user can be oriented at the desired height and HUT angle. A custom back‐support plate is situated on top of the plinth and allows for the suspension of the user's pelvis and thorax segments. The plate currently weighs 16.4 kg and comprises two aluminium sheets, with foam padding for the hip and trunk/shoulder regions, connected to each other by a universal joint allowing six degrees of freedom (DOF) motion between the pelvis and thorax body segments.

The ankle and knee suspension ropes connect to slings that provide support under the foot/ankle and knee, respectively. The centrally located suspension ropes for the thorax and pelvis attach to a custom hanger which allows a rope to attach to the left and right sides of the back‐support plate, whilst allowing axial rotation of each of these body segments. Eye bolts located on the outside of the back‐support plate allow for the ropes attached at their highest point to the left and right sides of the pelvis and thorax hangers to be connected to the back‐support plate using carabiners.

The VGSS plate or ‘floor’ surface, which the feet are in contact with when the body is in the initial equilibrium position, is oriented perpendicular to the axial line of the body (i.e., vertical in horizontal suspension or inclined at the same angle as the body‐suspension angle, albeit perpendicular to the body in HUT suspension). This ‘floor’ surface comprises a motorized treadmill (Floatride, Reebok, Boston, MA, USA) mounted on two force platforms (OR6‐7, AMTI, Watertown, MA, USA), themselves mounted on a 10 mm‐thick aluminium plate which is connected to the VGSS frame via a 16 mm‐diameter stainless steel bar passing through pillow bearings attached to the rear of the aluminium plate and the VGSS frame, allowing the ‘floor’ surface to be angled with respect to vertical in order to achieve the inclinations required for hypogravity simulation. The floor surface can be tilted in a controlled manner via a pair of linear actuators (LA60, Servo Components & Systems Ltd, Poole, UK). For level locomotion in simulated hypogravity, the axial line of the participant's body must remain perpendicular to the inclined floor surface. However, the VGSS can also support uphill or downhill locomotion by adjusting the body's axial alignment to match the desired hypogravity condition (e.g., 9.5° for lunar gravity) while independently setting the floor surface to a steeper angle for downhill or a shallower angle for uphill movement. The VGSS allows the floor surface to be inclined between 0° and 30°.

Suspension ropes from each body segment travel upwards toward a series of overhead gantries. Each gantry is comprised of an aluminium strut profile situated on a pair of sliding elements and has either a centrally located pulley (thorax and pelvis segments) or a pair of pulleys (i.e., one above each leg) which direct the rope towards the rear of the VGSS. The ankle gantry forms part of a larger frame which the knee, pelvis and thorax gantries can move along. This frame can be moved by a pair of linear actuators (LA60, Servo Components & Systems Ltd, Poole, UK). The remaining three gantries are each moved by a pair of linear actuators (LA35, Servo Components & Systems). The position of each gantry is set to allow for the inclination of each rope to be perpendicular to the body's axial line when the body is in its initial equilibrium.

Each rope is directed by its corresponding pulley towards the rear VGSS frame, within which a series of spring balances (YBF series, Yale, Greenville, NC, USA) are located, that apply a force to each rope equal to the weight of its corresponding body segment to counterbalance each body segment. The constant load applied to each rope by its corresponding spring balance can be manually adjusted within the loading range of the spring balance. Due to the spring balances each having a limited loading range, they are interchangeable within the VGSS, such that the appropriate load‐range spring‐balance can be used, depending on the participant's body segment masses. The spring balance cables can each be lowered in a controlled manner by a manual winch to set each body segment to the desired starting position. Once at the desired starting position, each body segment's respective spring balance is adjusted to set the loading applied to the suspension rope and, therefore, the body segment, to match the weight of the body segment. Once the spring balance loading is set to the required load for each participant and the respective body segment, the winch is removed, leaving the body segment suspended in a counterbalanced state. When suspended, each body segment is free to move in the anteroposterior direction through its respective suspension rope shortening or lengthening. This, therefore, allows for any forward lean of the body that is typically seen in some ambulatory activities (De Witt et al., 2010). The spring balances also enable their rope to be locked at a certain length, if desired, in order to constrain anteroposterior motion of the corresponding body segment.

The weight of each body segment can be estimated using standard anthropometric models (e.g., (De Leva, 1996; Pavol et al., 2002; Plagenhoef et al., 1983; Winter, 2009) and subsequently fine‐tuned by the operator such that when the body segment is moved up or down from its original position either manually or by the participant, similar levels of force are required (determined subjectively or measured via a force gauge) and the body region does not fall or rise passively from its suspended position. Due to the constant‐load nature of the spring balances, once the correct counterbalancing load is set for each body segment, the participant can move each body segment in an anterior or posterior direction easily, as the spring balance counteracts the vertical gravitational acceleration acting on each body segment. The VGSS has a safe working load of 125 kg and no height restriction.

### 2.2 Theoretical framework

In horizontal suspension, the suspension ropes are initially vertical. In this condition, there is a net longitudinal (head‐to‐foot) acceleration of zero when the body is in its initial position or equilibrium, with the feet just in contact with a vertical plate. In HUT suspension, the body is tilted at an angle with respect to horizontal in a head‐up orientation and the suspension ropes are perpendicular to the tilt angle of the body when the body is in its initial equilibrium position with the feet resting on an inclined plate that is oriented perpendicular to the axial line of the body. The angle of suspension, θ_suspension_, dictates the net axial acceleration, *a*_axial_, acting to accelerate the body towards the inclined plate according to:

(1) $$a_{\mathrm{axial}}=g\sin\theta_{\mathrm{suspension}}$$

where *g* is Earth's vertical gravitational acceleration (Figure 1). For this technical report, a HUT angle of 9.5° was used, simulating the 0.17 *g* environment of the Moon, in which the body will experience a small net axial acceleration of −1.62 m s^−2^ when the feet are in contact with the inclined surface and the body is in equilibrium.

#### 2.2.1 Pendulum effect in horizontal suspension

**FIGURE 3:**
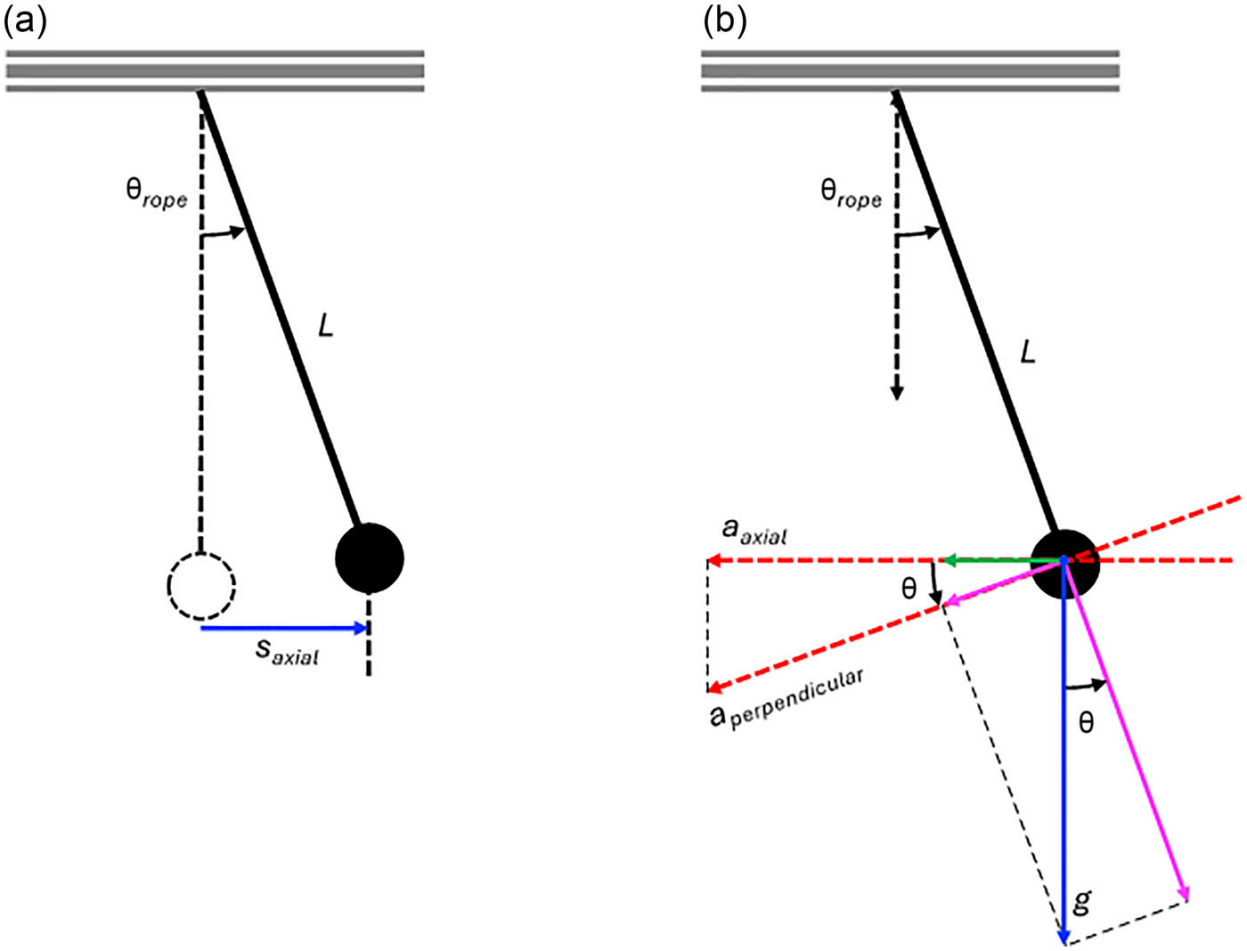
The body represented as a point mass suspended from a single rope or pendulum.

When the body is at equilibrium in horizontal suspension, Equation (1) dictates that the body will experience a 0 *g* axial acceleration. The body is initially aligned parallel to the ground with all suspension ropes vertical and of equal length, *L* (i.e., the distance from the participant's body to each rope's ceiling pulley). When the participant's body is displaced in the axial direction away from its initial equilibrium position (e.g., during jumping away from the vertical plate), the ropes displace by an angle, θ_rope_, away from vertical. As the suspension ropes are of equal length, assuming that the body acts as a rigid link, where the horizontal distance between each rope remains constant, the system can be modelled as a point mass suspended by a single rope (i.e., a simple pendulum), with this point‐mass remaining in a horizontal orientation independent of rope angle. The angular displacement of the rope, θ_rope_, is proportional to the rope length, *L*, and the linear axial displacement of the body, *s*_axial_ (Figure 3a), such that:

(2) $$\theta_{\mathrm{rope}}=\sin^{-1}\frac{s_{\mathrm{axial}}}{L}\;$$

Modelling the system as a simple pendulum, Earth's vertical gravitational acceleration, *g*, acts on the body to restore it to its equilibrium position, with this restoring acceleration increasing as the rope angle increases. It is important to understand how this restoring acceleration increases as a function of rope angle and, hence, linear axial displacement of the body, as it will act to increase the axial acceleration experienced by the body above that being simulated by the suspension system when the body is in its initial equilibrium position. The component of Earth's gravitational acceleration acting perpendicular to the rope, *a*_perpendicular_ (Figure 3b), is given by:

(3) $$a_{\mathrm{perpendicular}}=g\sin\theta_{\mathrm{rope}}$$

It then follows that the component of this perpendicular acceleration in the body's axial direction, *a*_axial_, is:

(4) $$a_{\mathrm{axial}}=g\sin\theta_{\mathrm{rope}}\cos\theta_{\mathrm{rope}}$$

**FIGURE 4:**
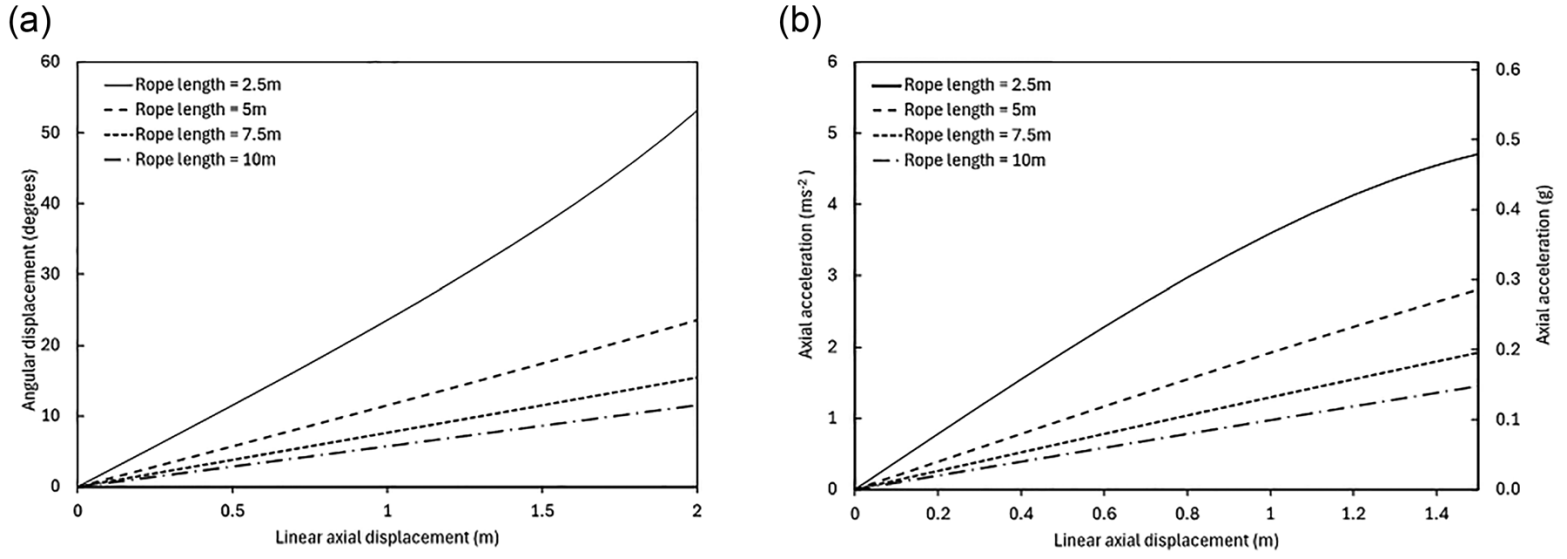
(a) Angular displacement (degrees), θ, of each rope as a function of linear axial displacement (m) of the body while suspended in the horizontal suspension condition. Data are shown for rope lengths of 2.5, 5, 7.5 and 10 m. (b) Axial acceleration (primary axis: m s^−2^; secondary axis: *g*), *a*, acting on the body as a function of linear axial displacement (m) of the body while suspended in the horizontal suspension condition. Data are shown for rope lengths of 2.5, 5, 7.5 and 10 m.

In horizontal suspension, *the axial force* should equal zero. As such, any increase in *a*_axial_ above zero due to the pendulum effect will act to accelerate the body back towards equilibrium and, therefore, introduce an error in the system which should be taken into consideration during data interpretation when dynamic movements (e.g. jumping) are performed in the VGSS. Figure 4 shows the relationship between linear displacement of the body in the axial direction and the resulting angular displacement of the ropes (Figure 4a) and the relationship between the restoring axial acceleration and linear axial displacement of the body away from equilibrium (e.g. during jumping) (Figure 4b). Data are presented for four rope lengths, illustrating a reduction in the angular displacement of the ropes as rope length is increased and a corresponding reduction in the axial component of Earth's gravity acting on the body due to the pendulum effect. These graphs illustrate the importance of suspending the body using ropes that are as long as possible, to minimise any deviation of the ropes from their initial vertical orientation, thus reducing the pendulum‐effect error acting to increase the simulated *g*‐level above the target condition.

#### 2.2.2 Pendulum effect in HUT suspension

**FIGURE 5:**
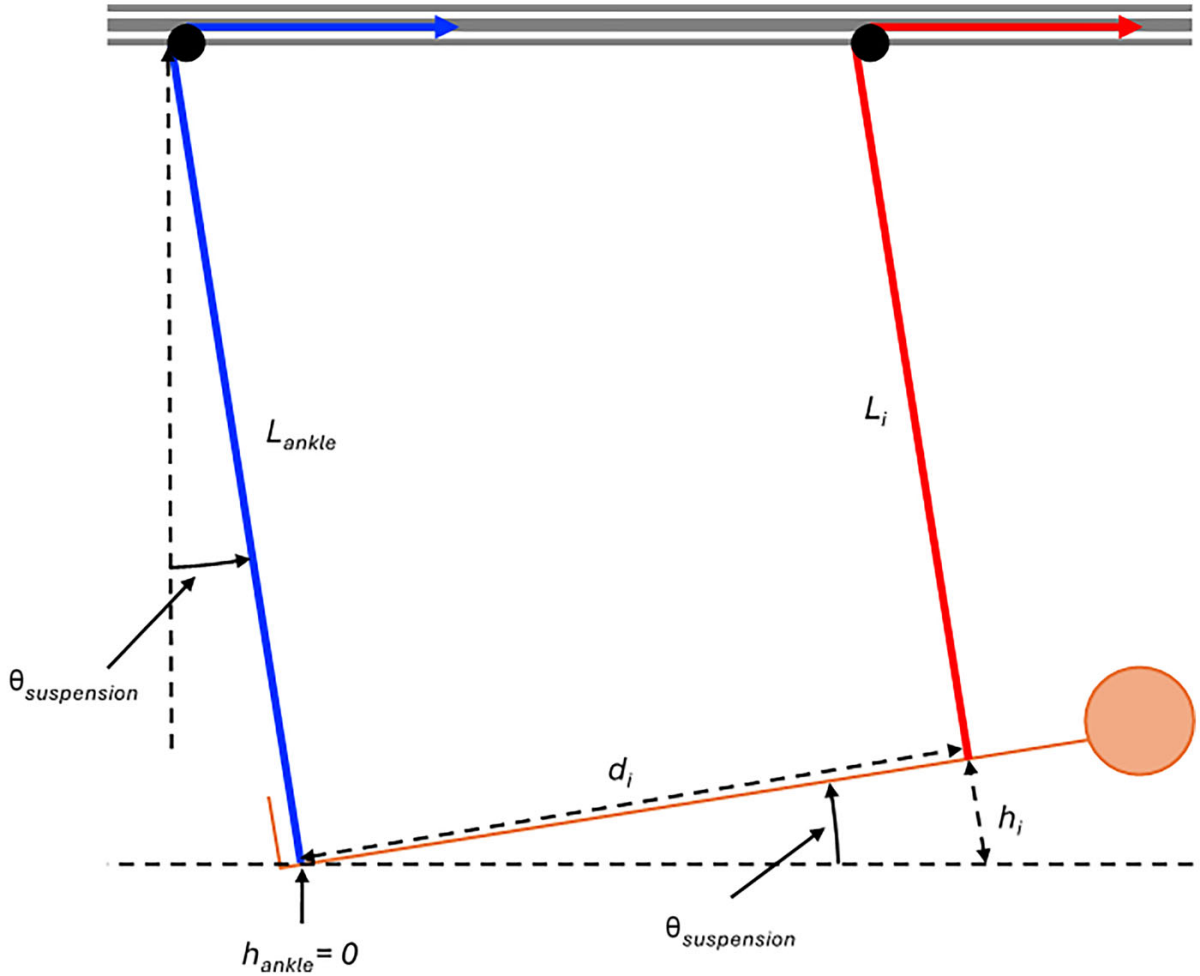
A body in 9.5° head‐up tilt suspension, illustrating the relevant variables for calculating the axial acceleration acting on each body segment above that experienced due to the inclination angle of the body in the equilibrium position.

During HUT suspension, the length of each rope between its attachment to each body segment and the top pulley, *L_i_*, will reduce as each body segment, *i*, is suspended progressively higher than the ankles, due to (1) the inclined head‐up orientation of the body and (2) the fixed height of the pulleys at the top of the VGSS that direct each rope towards the rear frame that houses the spring balances (Figure 5). As such, the body cannot be modelled as a point mass suspended from a single rope, as was possible for horizontal suspension. Instead, each rope and its corresponding body segment must be treated independently in order to determine the overall axial acceleration that is superimposed on top of the axial acceleration experienced in the equilibrium position due to the inclination of the body suspension.

The length of each suspension rope, *L_i_*, is proportional to the ankle rope length, *L*_ankle_ and the height of each body segment's rope attachment, *h_i_*, with respect to the ankle rope attachment to the body, perpendicular to the rope inclination, such that:

(5) $$L_{i}=L_{\mathrm{ankle}}\;-h_{i}$$

In the initial equilibrium position, the angle of all ropes will be equal to the angle of inclination, θ_suspension_. The relative height of each rope's attachment to its respective body segment, *h_i_*, is therefore given by:

(6) $$h_{i}=d_{\mathrm{axial},i}\;\tan\theta_{\mathrm{suspension}}$$

where *d*_axial_ *_,i_* is the axial distance between the ankle rope attachment and each subsequent rope's attachment to its respective body segment, *i*. Using standard anatomical proportions as a function of body height, *H* (Winter, 2009), the height of the ankle rope attachment above the inclined plate that the feet rest on in the equilibrium position can be assumed to equal 0.039*H*. The height of each subsequent rope's connection to its respective body segment above the height of the ankle, *d*_axial_ *_,i_*, is then given by:

(7) $$d_{\mathrm{axial},\mathrm{knee}}=0.285H-0.039H$$

(8) $$d_{\mathrm{axial},\mathrm{pelvis}}=0.53H-0.039H$$

(9) $$d_{\mathrm{axial},\mathrm{thorax}}=0.81H-0.039H$$

**FIGURE 6:**
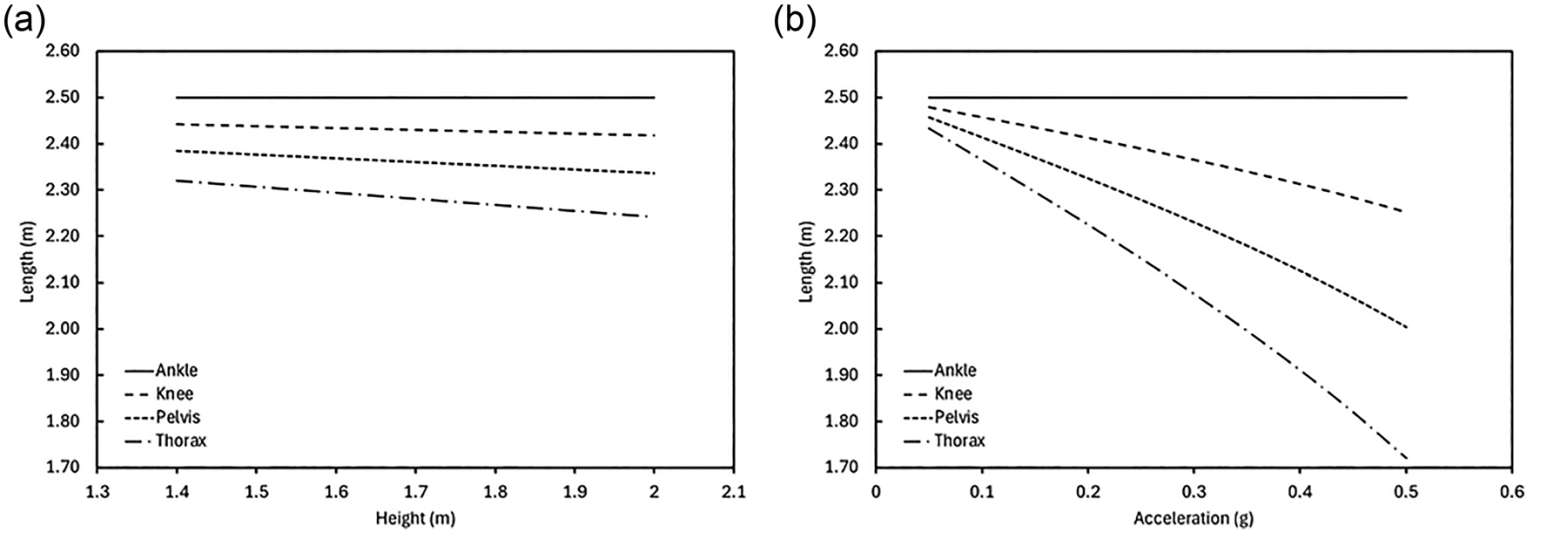
Rope length, *L_i_*, is shown for the ankle, knee, pelvis and thorax segments as a function of body height, *H*, for an example ankle rope length, *L*_ankle_, of 2.5 m during 9.5° head‐up tilt (a) and as a function of simulated gravitational acceleration, *g*, for an example body height, *H*, of 1.75 m (b).

Using Equations (5)–(9), assuming an ankle rope length of 2.5 m, Figure 6 presents the lengths of the knee, pelvis and thorax ropes as a function of participant height, *H*, in simulated lunar gravity (Figure 6a) and as a function of simulated axial acceleration, *g*, for a fixed height, *H*, of 1.75 m (Figure 6b). These data illustrate (1) how rope length reduces as the body segment moves progressively further from the ankle and (2) how rope length reduces to a greater extent between body segments as the simulated gravity (i.e., inclination angle of the body) increases.

**FIGURE 7:**
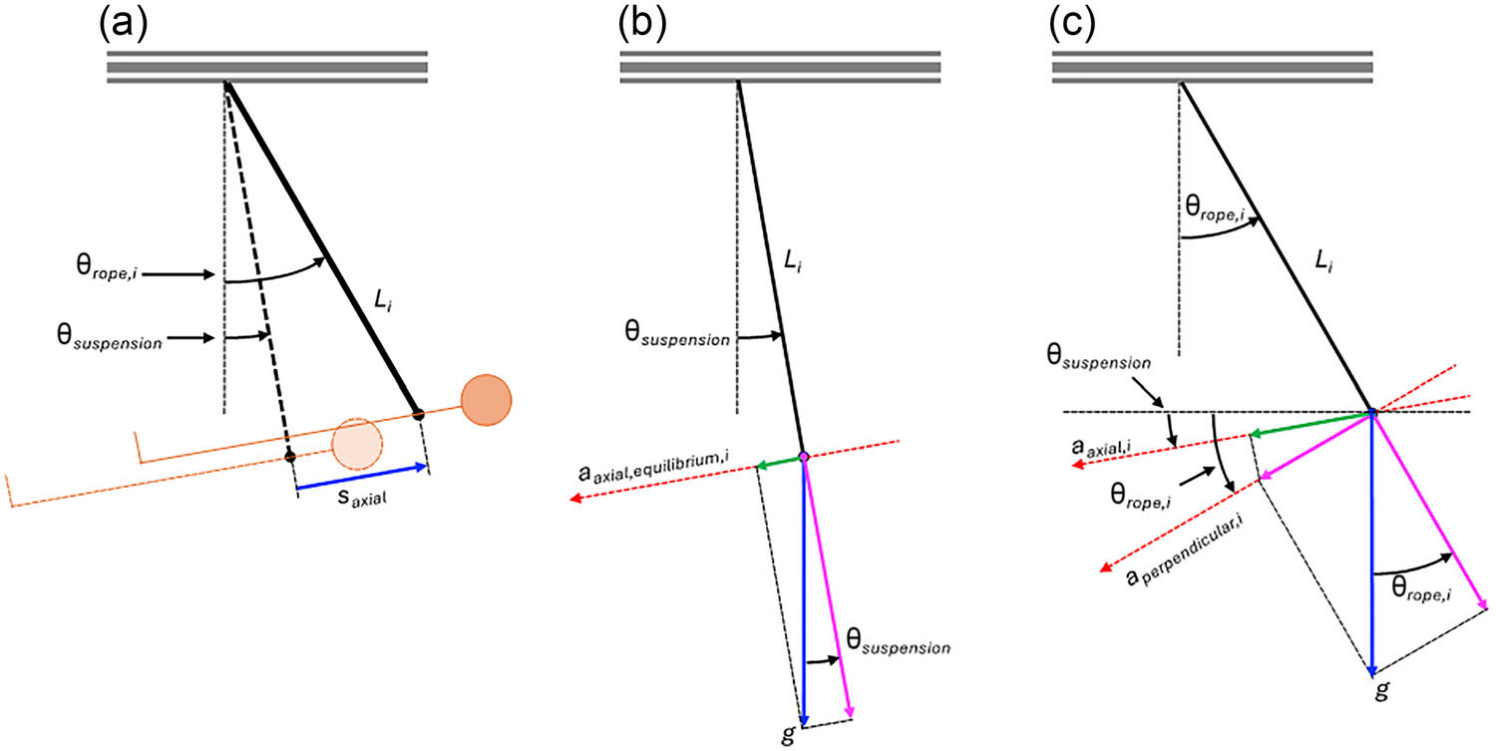
(a) Illustration of the angular and linear displacements of the suspended body and ropes. (b) The equilibrium position for a single rope, with the axial acceleration acting on the body equal to the perpendicular acceleration acting on the rope due to the rope being perpendicular to the body whilst in equilibrium. (c) A free body diagram for an example rope/body segment when the rope angle increases above the suspension angle.

**FIGURE 8:**
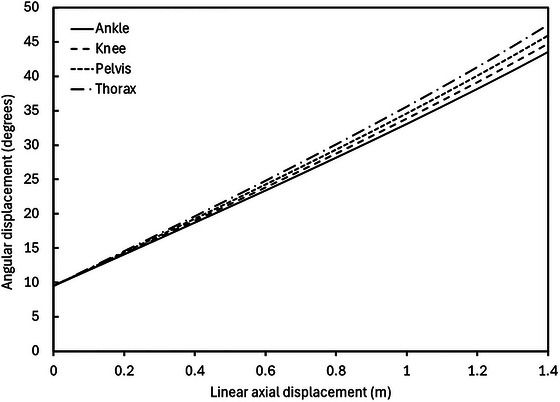
Angular displacement (degrees), θ, of the ankle, knee, pelvis and thorax ropes as a function of linear axial displacement (m) of the body in 9.5° (lunar gravity) head‐up tilt suspension. Body height is assumed to be 1.75 m for the modelled data shown.

Figure 7 presents a series of free‐body diagrams of the body in HUT suspension. As the ropes become progressively shorter for each body segment, *i*, further away from the ankle, the angular displacement of each rope from equilibrium, θ_rope_ *_,i_* (Figure 7a) will, for the same linear axial displacement of the body, *s*_axial_ *_,i_*, increase as a function of reduced rope length, *L_i_*. Assuming that the body moves as a rigid body so that the axial distances between each rope and the ankle rope remain constant, the angular displacement of each rope, θ_rope_ *_,i_*, is given by the following equation and illustrated in Figure 8 for simulated lunar gravity, assuming a body height of 1.75 m, as a function of linear axial displacement of the body:

(10) $$\theta_{\mathrm{rope},i}=\sin^{-1}\frac{s_{\mathrm{axial},i}}{L_{i}}+\theta_{\mathrm{suspension}}$$

In the equilibrium position in HUT suspension, each body segment will experience the same axial acceleration equal to the target simulated *g*‐level, *a*_axial,equilibrium_ *_,i_* (Figure 7b). As the ropes are all perpendicular to the body in equilibrium, it follows from Equation (3) that the axial acceleration acting on each body segment is:

(11) $$a_{\mathrm{axial},\mathrm{equilibrium},i}=g\sin\theta_{\mathrm{suspension}}$$

When the body is displaced away from equilibrium by a linear axial displacement, *s*_axial_, an additional axial acceleration will act on the body due to the pendulum effect (pendulum‐effect error). Due to the different rope lengths in HUT suspension, this additional axial acceleration must be considered separately for each rope. Equation (4) gives the axial acceleration acting on the body to restore it to a vertical equilibrium position in horizontal suspension. As the equilibrium position in HUT suspension is with the body inclined at the suspension angle, θ_suspension_, and assuming that the body stays at this inclination through the range of linear axial displacements through which the body moves away from equilibrium, the axial acceleration acting on each body segment, *a*_axial_ *_,i_* (Figure 7c), will be given by:

(12) $$a_{\mathrm{axial},i}=a_{\mathrm{axial},\mathrm{equilibrium}}\;\cos\left(\theta_{\mathrm{rope},i}-\theta_{\mathrm{suspension}}\right)$$

**FIGURE 9:**
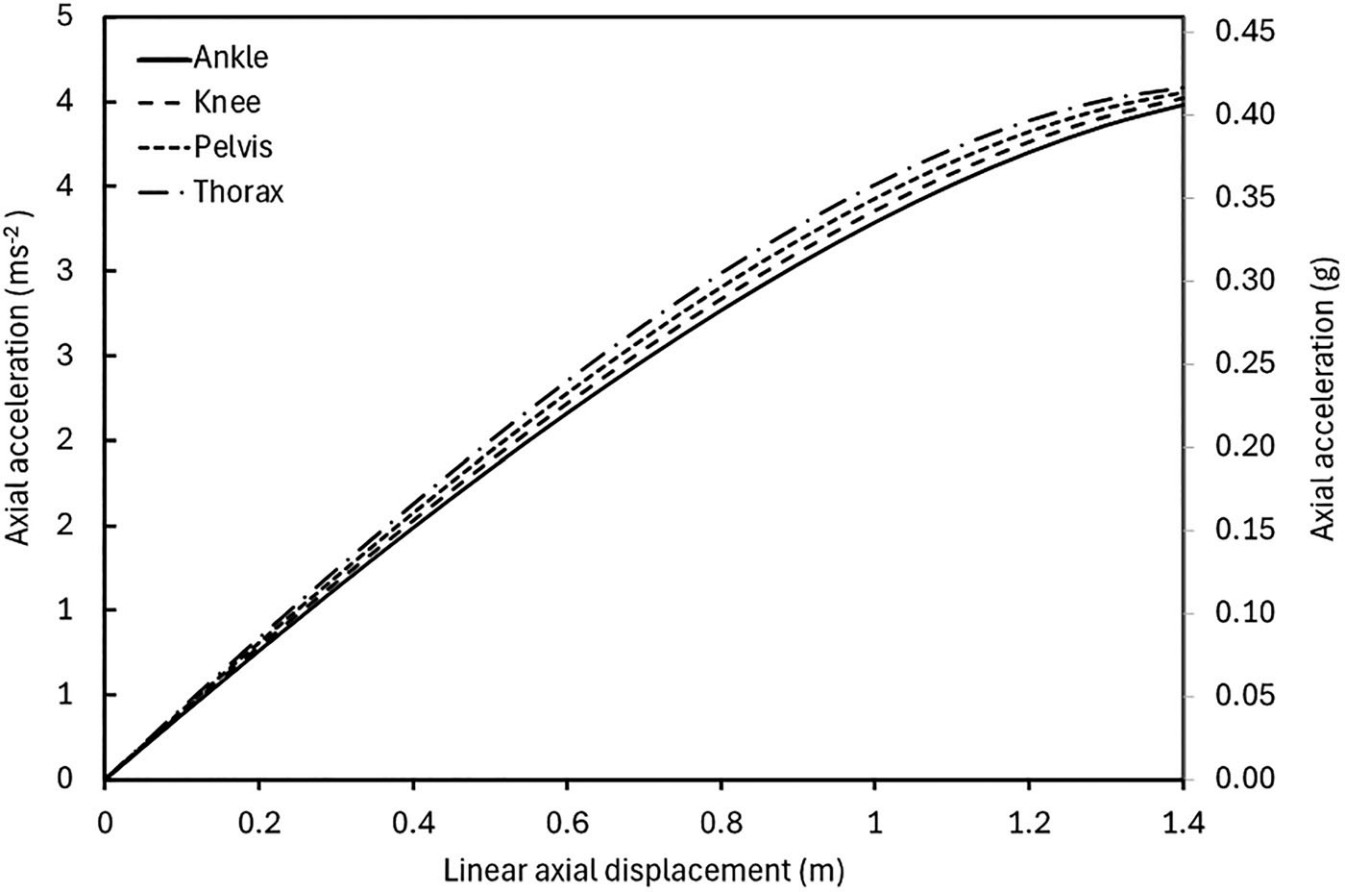
The additional axial acceleration (primary axis: m s^−2^; secondary axis: *g*), acting on the ankle, knee, pelvis and thorax body segments due to the pendulum effect of each of the corresponding suspension ropes is shown as a function of linear axial displacement (m) of the body while suspended at 9.5° head‐up tilt. The modelled data presented assume an ankle rope length of 2.5 m and a body height of 1.75 m for the initial calculation of the angular displacements of each of the four suspension ropes, from which axial accelerations are calculated.

Figure 9 illustrates the additional axial acceleration that acts on the body during HUT suspension (i.e. *a*_axial_ *_,i_*—*a*_axial,equilibrium_ *_,i_*) as a function of linear axial displacement of the body, due to the pendulum effect. The differences in this additional axial acceleration for each rope, resulting from the shorter rope lengths as the rope moves further from the ankle, can be seen. It is important to consider this pendulum‐effect error, which acts to increase the axial acceleration above the target simulated *g*‐level with linear axial displacement of the body away from the inclined plate, when interpreting data from any dynamic movements in HUT suspension.

The total axial acceleration of the participant's body above the simulated *g*‐level acceleration, *a*_axial,total_ is then found by considering the mass‐weighted sum of each body segment's acceleration calculated by Equation (12), with:

(13) $$a_{\mathrm{axial},\mathrm{total}}=\sum_{i=1}^{4}\left(\frac{m_{i}}{M}a_{\mathrm{axial},i}\right)$$

where *M* is the total body mass and *m_i_* is the body segment mass. By assuming anatomical proportions for body segment masses as a function of total body mass (Winter, 2009), the total mass‐weighted axial acceleration acting on the body.

### 2.3 Auxiliary hardware

The VGSS houses a range of biomechanical hardware in addition to the two force platforms that the treadmill is mounted onto. Signals from the two force platforms pass to strain gauge amplifiers (AMS‐6, AMTI). Each spring balance providing the counterbalance loads for each suspension rope is suspended beneath an s‐beam load cell (614, RS Pro, Corby, Northamptonshire, UK) which has a stated linearity of 0.3% and hysteresis of 0.3%. Signals from each load cell pass to a strain gauge amplifier (custom built). The position of each gantry is determined by measuring its displacement using a cable extension potentiometer (SP1‐50, Multicomp Pro, Leeds, UK). The inclination of the treadmill is measured using an inclination sensor (TMM55E‐PMH045, Sick, Hertfordshire, UK). All force platform, load cell and cable extension transducer signals are sampled by a modular data acquisition system (cDAQ‐9174, National Instruments, Austin, TX, USA) via three analogue input modules (NI‐9102, National Instruments). The inclination sensor signal is sampled by the same modular data acquisition system via an analogue current input module (NI‐9174, National Instruments). All data are then passed to a computer and sampled at 2000 Hz within a custom program (LabVIEW 2018, National Instruments) which visualises the data in real‐time and writes the data into a text file for later processing and analysis. A computer screen can be mounted horizontally onto the VGSS directly above the participant's head, such that the participant can visualise live performance data. For example, an additional cable extension transducer (SP1‐50, Multicomp Pro) mounted to the side of the treadmill with the end of the cable attached to the participant at waist level can measure hip displacement perpendicular to the treadmill surface (e.g. to measure jump height), and the participant feedback display can show these data along with a target jump height, thus providing real‐time participant feedback during exercise.

### 2.4 Standard operation procedure

The following standard operating procedure is followed for suspending a participant in the VGSS:

1. The user lies upon the hip‐to‐head back support plate situated on top of the electronic plinth.
2. Ankle and knee support slings are secured around the limbs of the user.
3. The plinth is adjusted to the desired height and tilt (verified via a digital inclinometer)
4. Any slack within the suspension ropes is removed via winches, and the spring‐balance loading is adjusted to the respective body segment weight.
5. The inclination of the suspension rope when the participant is in the initial equilibrium position is verified via a digital inclinometer.
6. The plinth is lowered, suspending the user in position.
7. The operator and participant then move each leg up/down to determine whether each spring balance tension requires adjustment.

Initial testing during development of the VGSS and the above standard operating procedure revealed that counterbalancing the pelvis and thorax body segments using spring balances was challenging and that flexion/extension between the thorax and pelvic body segments was difficult to simulate during upward HUT suspension. Users struggled to self‐correct trunk misalignment, and activities had to be paused whilst the operator assisted in adjusting the trunk back to a neutral position. To overcome this issue, for the purposes of initial proof‐of‐concept testing, the spring balances for the pelvis and thorax body segments were locked after the participant's body segments were at the desired position, such that they acted as fixed ropes. This allowed the upper body to remain in a neutral position and greatly improved the ability to perform dynamic tasks such as repetitive countermovement jumping.

### 2.5 Experimental protocol

#### 2.5.1 Manikin suspension

A preliminary study was performed on an early prototype of the VGSS to establish whether the suspension of a manikin at various HUT angles would induce the expected levels of axial loading on the manikin. A fully articulated nursing manikin (height: 1.62 m; mass: 27.9 kg) was repeatedly suspended 30 times for 1‐min periods at each of the following levels of simulated hypogravity (HUT angle): 0.05 *g* (2.9°), 0.10 *g* (5.7°), 0.15 *g* (8.6°), 0.165 *g* (9.5°) and 0.20 *g* (11.5°). An additional set of suspensions was completed at 10 randomly generated gravity levels between 0 and 0.2 *g* – 0.00 *g* (0°), 0.02 *g* (1.1°), 0.03 *g* (1.7°), 0.04 *g* (2.3°), 0.07 *g* (4.0°), 0.09 *g* (5.2°), 0.11 *g* (6.3°), 0.13 *g* (7.5°), 0.14 *g* (8.0°) and 0.20 *g* (11.5°) – performed twice on a single day and once on a separate day to examine the accuracy and within‐ and between‐day reliability of the entire suspension process.

The VGSS prototype did not yet have the custom back support plate, so a head‐to‐thigh lifting sling was used to support the body. It also did not use spring‐balancers, and therefore each of the suspension ropes was manually adjusted to the required length, such that the axial line of the manikin was at the desired HUT angle, subsequently being locked off by the operator using a clutch mounted at the rear of the VGSS. The HUT angle of the suspended manikin was then verified by measuring the angular displacement between the axial line (defined as the mid ear to the lateral malleolus) of the manikin with respect to horizontal using a photo taken in the sagittal plane and uploaded to the Angle Meter 360 app (Version 1.8). Vertical ground reaction force (vGRF) was measured via a single force platform (zeroed at every new tilt position prior to suspension of the manikin) mounted on the VGSS foot platform. The mean vGRF over the last 30 s of each trial was used for analysis and compared against the theoretically expected vGRF for the given HUT angle.

#### 2.5.2 Human suspension

##### Ethical approval

All participants provided written informed consent prior to participation. Ethical approval was granted by the Northumbria University Faculty of Health and Life Sciences Ethics Committee (reference codes: 46356, 6515 and 1993) and conducted in accordance with the 2024 *Declaration of Helsinki*, except for registration in a database.

##### Study design

The capability of the VGSS to simulate lunar gravity in humans was initially assessed by measuring the standing vGRF of participants suspended in a 9.5° HUT position. These data were collected as part of a larger study reported in full elsewhere (Swain et al., 2026). Fifteen healthy adults (age: 26 ± 4 years, height: 1.77 ± 0.08 m, mass: 77.3 ± 11.8 kg) completed a single visit to the laboratory. Participants’ mass was measured to within 0.1 kg (Seca 703 Digital Column Scale, Hamburg, Germany). Participants were suspended in simulated lunar gravity (9.5° HUT) and stood quietly for 30–60 s. Participants laid upon an electronic plinth tilted to 9.5° and with their feet in contact with the VGSS foot platform. The participant, whose pelvis and thorax body segments were supported by the custom back‐support plate described earlier, was suspended in this position by locking‐off the suspension ropes following removal of the rope slack, as spring balances had not yet been incorporated into the suspension mechanism of the VGSS, before the mean standing vGRF was measured in the same manner as for the manikin study. The accuracy and reliability of simulating lunar gravity within the VGSS were re‐assessed following the introduction of spring balancers into the system. Nineteen healthy adults (age: 24 ± 4 years, height: 1.80 ± 0.06 m, mass: 72.4 ± 10.1 kg) completed the first visit, 14 of whom (age: 24 ± 4, height: 1.81 ± 0.06 m, mass: 74.5 ± 11.8 kg) went on to complete a further two visits. These data were collected as part of two larger studies, one of which is reported in full elsewhere and the other is currently in preparation for publication (Swain et al., 2026). During each visit, the mass of the participant was measured before the participant was suspended in simulated lunar gravity (9.5° HUT) in which they stood quietly for 10–30 s to verify the level of weight‐bearing. Video recordings were taken of a user performing locomotion and jumping activities within the VGSS to showcase the system in operation.

### 2.6 Data processing

A custom MATLAB (MathWorks, Natick, MA, USA) program filtered the force data using either a 20 Hz low‐pass second‐order Butterworth filter (manikin experiments and human study 1) or a 50 Hz low‐pass fourth‐order Butterworth filter (human study 2). The level of partial weight‐bearing experienced by the manikin and participants in the first human study was computed as the standing vGRF expressed as a percentage of body weight measured on the day of testing. In the second human study, to which the main objective was related to the metabolic effects of jumping in hypogravity, it became apparent that some participants experienced some axial weight‐bearing in the anterior–posterior direction as measured by the force platform, due to the position of the foot and leg not being entirely perpendicular to the foot platform during take‐offs/landings. To account for this, the resultant of the vertical and anterior–posterior ground reaction forces (*F*_z_ and *F*_y_) was used to compute the level of partial weight‐bearing, including during the initial standing phase reported in the present document.

The reliability of weight‐bearing between retest suspensions for the manikin data was assessed using intra‐class correlation coefficients (ICC) (model: two‐way mixed effects; type: absolute agreement) with 95% confidence intervals (CI); ICC values were described according to the following thresholds: <0.50 (poor), 0.50–0.74 (moderate), 0.75–0.90 (good) and ≥0.90 (excellent) (Koo & Li, 2016). Statistical significance was set at *P* < 0.05. Statistical analysis was performed using SPSS Statistics (Version 27; IBM Corp., Armonk, NY, USA). Blant–Altman plots were used to assess the relative bias (mean difference) and limits of agreement (LOA: 1.96 standard deviations of the difference) for standing vGRF in participants that were suspended in simulated lunar within the VGSS on three separate days. The 95% CIs were calculated to examine the estimation uncertainty for bias and LOAs. Data are presented as mean ± standard deviation.

## 3 RESULTS

### 3.1 Manikin suspension

The relationship between weight‐bearing and simulated gravity level during manikin suspension at a range of suspension angles was linear (*R* ^2^ = 0.96) (Table 1). The mean difference between the target and measured vGRF (% bodyweight) was 1.8 ± 0.9% (range: 0.4–3.0%). The foot plate angle could be precisely angled to within 0.1° of the target position, and the orientation of the manikin could be accurately suspended perpendicular to the foot plate (Table 1). Data for the suspension of the manikin at randomly generated gravity levels between 0 *g* and 0.20 *g* are presented in Table 2. Suspensions at 0.02 *g* were excluded as the manikin failed to contact the force platform. The mean difference between the expected and measured vGRF (% bodyweight) was 0.8 ± 1.6% (range: −1.0 to 4.0%). ICCs were excellent (>0.90) for within‐ and between‐day test–retests. The ICCs were 0.99 (95% CI: 0.97–0.99, *P* < 0.0001) for within‐day trials (test 1 vs. 2) and 0.99 (95% CI: 0.99–1.00, *P* < 0.0001) and 0.99 (95% CI: 0.98–0.99, *P* < 0.0001) for the between‐day test 1 versus 3 and test 2 versus 3, respectively. Differences in weight‐bearing between retests were within ±2% bodyweight.

**TABLE 1:** Simulated microgravity and hypogravity suspension angle and vertical ground reaction force (*n* = 30 per condition).

Gravity level	Foot plate angle (°)	Manikin orientation (°)	Target load (% bodyweight)	Measured load (% bodyweight)
0.00 *g*	90.0 ± 0.0	89.7 ± 0.5	0.0	2.0 ± 0.6
0.05 *g*	87.1 ± 0.0	89.8 ± 1.0	5.0	5.4 ± 0.8
0.10 *g*	84.3 ± 0.0	90.0 ± 0.9	10.0	11.1 ± 0.5
0.15 *g*	81.4 ± 0.0	89.5 ± 1.0	15.0	17.0 ± 0.7
0.17 *g*	80.5 ± 0.0	89.8 ± 1.0	17.0	18.4 ± 0.9
0.20 *g*	78.5 ± 0.0	90.0 ± 0.9	20.0	23.0 ± 0.8

*Note*: Manikin longitudinal axis orientation is relative to the foot platform.

**TABLE 2:** Suspension angles and vertical ground reaction force for simulations performed at randomly generated gravity levels between 0 and 0.20 *g* (*n* = 3 per condition).

Gravity level	Foot plate angle (°)	Manikin orientation (°)	Target load (% bodyweight)	Measured load (% bodyweight)
0.00 *g*	90.0 ± 0.0	90.0 ± 0.0	0.0	2.0 ± 0.1
0.03 *g*	88.3 ± 0.0	89.3 ± 0.6	3.0	2.1 ± 0.4
0.04 *g*	87.7 ± 0.0	89.0 ± 1.7	4.0	3.0 ± 0.6
0.07 *g*	86.0 ± 0.0	87.7 ± 0.6	7.0	6.5 ± 0.3
0.09 *g*	84.8 ± 0.1	91.3 ± 1.5	9.0	9.4 ± 0.5
0.11 *g*	83.7 ± 0.0	88.7 ± 1.2	11.0	12.0 ± 0.2
0.13 *g*	82.5 ± 0.0	90.3 ± 1.2	13.0	13.8 ± 0.7
0.14 *g*	82.0 ± 0.1	90.3 ± 1.2	14.0	15.4 ± 1.1
0.20 *g*	78.5 ± 0.0	91.0 ± 1.0	20.0	24.0 ± 0.9

*Note*: Manikin longitudinal axis orientation is relative to the foot platform.

### 3.2 Human suspensions

Table 3 displays data from the first human study in which the level of weight‐bearing during standing was recorded in *n* = 15 participants upon being suspended in simulated lunar gravity (9.5° HUT). Note that due to the participants lying on a back support (lunar mass = 2.7 kg) whilst suspended, this additional mass was included in the actual lunar body mass measurement, as the back support mass contributes to weight‐bearing in addition to the participant's own body mass. Participants experienced a mean vGRF of 19.6 ± 1.9% bodyweight (range: 17.1–23.6%) with a mean error from the target load (i.e., 16.5% bodyweight) of 3.1 ± 1.9% bodyweight.

**TABLE 3:** Standing weight‐bearing in simulated lunar gravity (0.165 *g*) – Study 1.

Participant	Earth body mass (kg)	Measured lunar body mass (kg)	Lunar loading (% bodyweight)	Error (% bodyweight)
1	69.8	13.2	19.0	2.5
2	79.7	13.6	17.1	0.6
3	77.6	17.6	22.7	6.2
4	96.2	18.7	19.4	2.9
5	79.0	15.5	19.6	3.1
6	95.6	19.3	20.2	3.7
7	68.8	12.1	17.6	1.1
8	65.2	11.2	17.2	0.7
9	87.4	18.8	21.5	5.0
10	86.1	16.4	19.1	2.6
11	74.7	14.4	19.2	2.7
12	66.7	13.2	19.9	3.3
13	71.1	16.8	23.6	7.1
14	54.0	9.7	18.0	1.5
15	87.4	17.8	20.4	3.9
Mean ± SD	77.3 ± 11.8	15.2 ± 3.0	19.6 ± 1.9	3.1 ± 1.9

*Note*: Error = actual lunar loading minus lunar gravity (0.165 *g*) – target load. Actual lunar body mass includes both participant mass and the back support mass, as both contribute to the weight‐bearing load.

Table 4 displays data from the second human study in which *n* = 19 participants were suspended in simulated lunar gravity on a single occasion, and a subgroup of *n* = 14 were suspended at the same gravity level on two further occasions, all on separate days. The mean level of weight‐bearing (% bodyweight) was 17.3 ± 2.8% (visit 1), 19.1 ± 3.0% (visit 2) and 17.6 ± 2.2% bodyweight (visit 3). The mean error between the observed and target vertical weight‐bearing (% bodyweight) was 0.8 ± 2.8% (visit 1), 2.6 ± 3.0% (visit 2) and 1.1 ± 2.2% (visit 3).

**TABLE 4:** Standing weight‐bearing in simulated lunar gravity (0.165 *g*) on three separate days – Study 2.

Participant	Earth bodyweight^a^ (kg)	Lunar loading (% bodyweight)			Error (% bodyweight)		
		Visit 1	Visit 2	Visit 3	Visit 1	Visit 2	Visit 3
1	64.6	17.6	18.7	18.0	1.1	2.2	1.5
2	69.0	20.8	19.1	17.4	4.3	2.6	0.9
3	68.7	16.3	NA	NA	−0.2	NA	NA
4	74.1	19.3	26.0	18.7	2.8	9.5	2.2
5	73.9	19.1	NA	NA	2.6	NA	NA
6	65.2	22.8	17.6	16.6	6.3	1.1	0.1
7	72.0	14.4	20.9	20.3	−2.1	4.4	3.8
8	62.9	16.9	18.3	20.6	0.4	1.8	4.1
9	77.8	16.5	16.4	16.0	0.0	−0.1	−0.5
10	60.6	17.5	NA	NA	1.0	NA	NA
11	59.6	19.3	16.6	19.8	2.8	0.1	3.3
12	59.4	18.3	NA	NA	1.8	NA	NA
13	76.0	14.2	NA	NA	−2.3	NA	NA
14	92.9	20.3	16.3	13.2	3.8	−0.2	−3.3
15	77.1	11.8	24.6	20.4	−4.7	8.1	3.9
16	66.5	14.5	20.5	15.6	−2.0	4.0	−0.9
17	87.5	15.6	16.4	14.8	−0.9	−0.1	−1.7
18	100.0	19.9	17.2	18.0	3.4	0.7	1.5
19	74.4	14.3	19.1	17.5	−2.2	2.6	1.0
Mean ± SD	72.4 ± 10.1	17.3 ± 2.8	19.1 ± 3.0	17.6 ± 2.2	0.8 ± 2.8	2.6 ± 3.0	1.1 ± 2.2

^a^ Bodyweight at the first visit is reported. Lunar weight‐bearing was normalised to the bodyweight of the participant on the day of each visit. NA: did not participate in visits 2 and 3. Error = actual load − target load (16.5% bodyweight).

**FIGURE 10:**
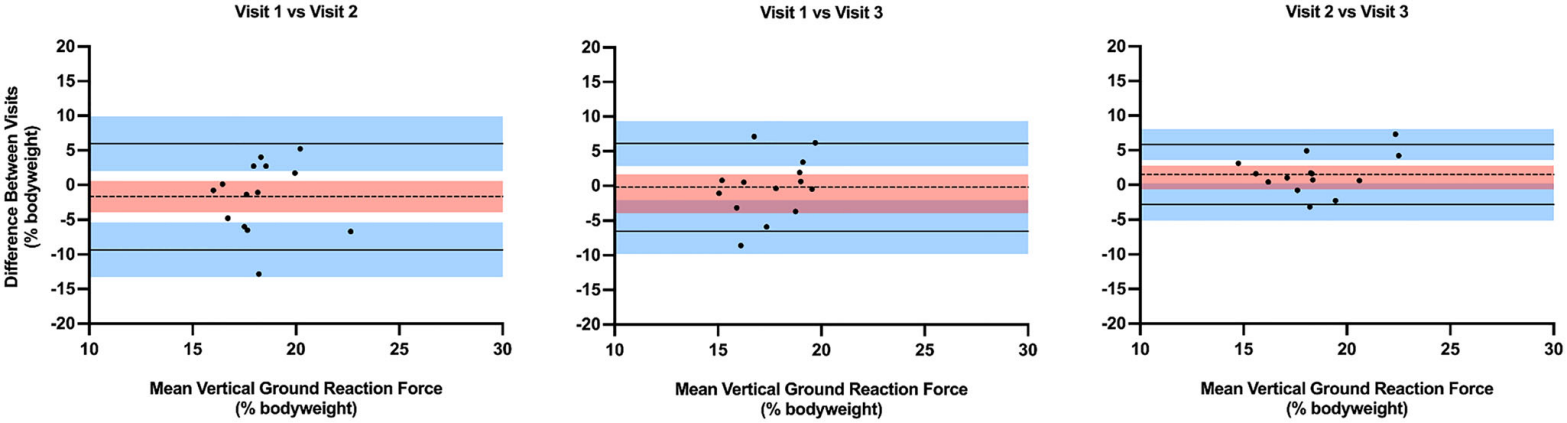
Bland–Altman plots presenting the mean standing vertical ground reaction force (*x*‐axis) against the mean difference in standing vertical ground reaction force (*y*‐axis) during suspension between the three laboratory visits (*n* = 14). Dashed lines represent the average mean difference for each comparison. Continuous lines represent the upper and lower 95% limits of agreement. The red and blue shaded areas reflect the 95% confidence intervals for the estimations.

Figure 10 displays Bland–Altman plots for standing vGRF (% bodyweight) in participants who were suspended in VGSS in simulated lunar gravity on three separate days. The mean bias and 95% CIs were: −1.7% (95% CI: −3.9, 0.6%), −0.2% (95% CI: −2.1, 1.6%) and 1.5% (0.2, 2.8%) for visit 1 versus visit 2, visit 1 versus visit 3, and visit 2 versus visit 3, respectively. The upper and lower LOAs for the respective comparisons were: 6.0% (2.0, 9.9%) and −9.3% (−13.3, −5.4%); 6.1% (2.8, 9.4%) and −6.5% (−9.8, −3.3%); and 5.8% (3.6, 8.0%) and −2.9% (−5.1, −0.6%).

## 4 DISCUSSION

### 4.1 Proof‐of‐concept findings

This technical report introduced the VGSS, a recently developed hypogravity analogue within the UK to support human movement in hypogravity research. Proof‐of‐concept findings demonstrated that the VGSS can suspend a body at variable levels of partial weight‐bearing, thus simulating hypogravity. Human participants suspended within the VGSS at simulated lunar gravity (0.17 *g*) experienced on average ∼17–19% bodyweight loading. On average, the error between the target and actual load was ±1–3% bodyweight, and the mean difference between repeat suspensions on separate days was ±1–2% bodyweight. Readers are directed to Supporting Videos 1 and 2 to view footage of jumping and locomotion performed in Simulated Lunar gravity within the VGSS, respectively.

### 4.2 Bodyweight support advantages and limitations

Each suspension analogue (vertical, horizontal and HUT) has unique advantages and limitations that it is prudent to acknowledge when considering their application in research and other contexts (e.g., astronaut training). Fundamentally, the DOF enabled by each suspension analogue can differ, and each may have specific restrictions on movement. NASA's ARGOS employs vertical suspension by providing a controlled upward force on a user‐worn harness via a human‐rated robotic system within a large test area, which enables dynamic human movement in *X*, *Y*, *Z* translational DOF to facilitate a wide range of activities (Bekdash et al., 2020). Understanding ‘unsuited’ (i.e., shorts and T‐shirt) human biomechanics in hypogravity is valuable for addressing the design of habitats and spacesuits for surface extra‐vehicular activities (EVAs). A key feature of surface exploration missions will be suited EVAs in which it is important to address the specific biomechanics of movement in a spacesuit due to the unique physical constraints they can impose. For instance, work by Carr and Newman concluded that the metabolic cost of transport is elevated during spacesuit activity, that it would likely be more metabolically efficient to run than to walk in a spacesuit within simulated gravitational environments, and that there may exist an optional spacesuit leg stiffness for a given level of gravity (Carr & Newman, 2007). Vertical suspension does benefit from its simplicity in integrating spacesuit components that offer a high‐fidelity simulation of surface EVAs (e.g., Norcross et al., 2010), and the VGSS system design is flexible enough to be able to accommodate spacesuit components.

Analogues in which the body is suspended in a horizontal or HUT position allow freedom of movement in the sagittal plane, whilst maintaining the target reduced gravity condition. Though lateral and rotational movements may be technically possible within these analogues, deviations of the orientation of the moving body segment(s) with respect to gravity likely violate the simulation and may also create artificial movement patterns due to the influence of the suspension apparatus, such as the ropes, causing pendular motion (e.g., during lateral movement). In addition, the tendency for balance is likely reduced during horizontal or HUT suspension due to the support provided by the hip‐to‐head cradle, as a large component of gravity acts along the anteroposterior axis. This makes it difficult or even impossible for the user to fall over or perform certain tasks such as forward leaning and kneeling, which are typical movements performed during EVAs.

Orienting the body on its side during HUT suspension can minimise these restrictions by allowing the ‘large’ component or remainder of gravity to act along the medio‐lateral axis. Video footage of NASA's RGWS (sideward HUT suspension) shows users’ balance and limb coordination being challenged during various movements (e.g., locomotion and jumping), including falling over and attempting to stand once fallen (NASA, 1965). Suspension analogues that allow the user to move along a walkway or within a test volume, allow the user to control the momentum of the body/limbs, which can more accurately mimic the demands of movement in actual hypogravity. However, a potential limitation to freely displacing the body is the influence of inertia imparted to the body from the lifting/suspension mechanism that must follow the user.

The operational volume in which the user performs activities can limit the scope of tasks that can be performed or restrict movement capability. Weber and colleagues investigated hopping in hypogravity using the European Space Agency's Vertical Treadmill Facility (horizontal suspension with a subject‐loading device) that could only facilitate a maximum hop height of 0.2 m due to headroom restrictions (Weber et al., 2019). The larger VGSS allows jumping up to 0.7 m, and NASA's Reduced Gravity Walking Simulator appeared not to have a headroom limit, enabling users to freely jump as high as possible. For reference, it is estimated that jump heights of ∼4 m are possible in lunar gravity (Cavagna, 1972).

Recognising the influence of pendulum swinging in horizontal/HUT suspension is crucial when discussing jump height and headroom limits between suspension analogues. Suspending the body using fixed ropes causes the body to conform to the path of a pendulum, the period of which is defined by the rope length. Whilst longer suspension ropes reduce pendulum swinging for the same jump height and more accurately simulate flight trajectories to those seen in actual hypogravity, a larger facility would be required. The consequences of suspending the body at the end of a rope or pendulum, becomes more pronounced with shorter rope lengths, as they will reduce vertical displacement and jump height for a given impulse leading to an increase in movement frequency during cyclical tasks (e.g., continuous bodyweight jumping) that may have implications for factors such as energy expenditure and fatigue. It is also worth considering whether the movement(s) of interest may be impeded by the straps/cables required for suspension. From our experience and as presented in Supporting Videos 1 and 2, the VGSS can accommodate locomotion and jumping activities without restriction.

A benefit of HUT suspension is that the lower limbs are subjected to reduced gravitational loading during both the stance and swing phases of gait (Sylos‐Labini et al., 2013). Vertical suspension off‐loads a target component of the user's centre of mass while the body is upright; therefore, the lower limb remains under a state of partial weight‐bearing during the stance phase of gait; however, within the swing phase, the leg is subject to 1 *g*. Gravity appears to play an important role in swing phase gait dynamics as Sylos‐Labini and colleagues identified that the leg swings faster and over a shorter distance during locomotion in vertical suspension analogue compared to tilted suspension, in which the leg swings slower and over a greater distance (Sylos‐Labini et al., 2013). Interestingly, relative to the 1 *g* control condition, longitudinal foot excursion was reduced during vertical suspension, whereas the opposite occurred during tilted suspension (Sylos‐Labini et al., 2013). The influence of gravity acting along the anteroposterior axis of body segments must, therefore, be considered for horizontal and HUT suspension.

HUT suspension offers a key advantage over horizontal suspension in simulating hypogravity, as loading experienced by the user is provided through a component of gravity as opposed to a subject‐loading device that provides a pull‐back force on the user using a harness. Subject‐loading devices (SLD) create areas of high pressure on the body at the shoulder and/or hip regions and can become uncomfortable at high loads or if worn for prolonged periods. In addition, the pull‐back force imparted onto the body during movements can be non‐constant to varying degrees depending on the loading device employed (e.g., bungees, springs, pneumatic cylinders) (De Witt et al., 2010; Weber et al., 2019). Notwithstanding these limitations, SLDs, alongside either horizontal or vertical suspension analogues, can permit the user to experience a wide range of partial weight‐bearing loads that can be particularly beneficial when studying the dose–response effects of gravity on human movement. Moreover, horizontal suspension with an SLD reproduces how astronauts walk and run on the International Space Station using the Combined Operational Load Bearing External Resistance Treadmill (T2‐Colbert) (De Witt et al., 2010).

The alignment of the body with respect to Earth's gravity alters hydrostatic pressure gradients in fluid‐filled bodily compartments, and head‐up/down body tilting can significantly impact cardiovascular haemodynamic and autonomic regulation (Hinghofer‐Szalkay, 2011; Whittle et al., 2022). Depending on the suspension analogue employed, the user can be situated in an upright, horizontal or supine/sideward HUT position. Head‐up tilting has the advantage of being able to alter the vertical hydrostatic pressure gradient by allowing a fraction of Earth's gravity to act along the axial line of the body and has been used to explore the cardiorespiratory responses to hypogravity (Baranov et al., 2016; Diaz‐Artiles et al., 2019; Pavy‐Le et al., 1997). The ‘other’ component of gravity acting either along the anteroposterior (in supine HUT suspension) or medio‐lateral (in sideward HUT suspension) axis must, however, be considered, as this will remain acting on fluids and tissues within the body that would otherwise not occur in real hypogravity. Researchers should thus remain mindful of how body inclination influences cardiovascular and respiratory function. Systemic circulation and cardiac control are highly sensitive to hydrostatic pressure changes during HUT and head‐down tilt (HDT) manoeuvres (Whittle et al., 2022). In HUT, fluids shift toward lower extremity vascular beds, reducing central blood volume, venous return, cardiac filling and left ventricular end‐diastolic pressure. This causes a decline in stroke volume via the Frank–Starling mechanism. The reduction in pressure on the arterial baroreflex decreases vagal tone and promotes sympathetic drive, leading to tachycardia and vasoconstriction, which raises heart rate and total peripheral resistance. These responses develop in a graded fashion with the degree of HUT and occur in reverse during HDT (Whittle et al., 2022).

The effect of posture extends to cardiorespiratory performance and the energetics of movement. Alterations in central blood volume, tissue perfusion pressures and pulmonary mechanics (e.g., displacement of the diaphragm and mediastinum and abdominal structures) must be recognised when interpreting data from bodyweight suspension analogues (Katz et al., 2018). When assessing the metabolic cost of a movement in hypogravity, such as jumping, it can be useful to normalise parameters like heart rate, minute ventilation, O_2_ consumption and CO_2_ production to peak values obtained from a cardiopulmonary exercise test (CPET), typically performed on a cycle ergometer in an upright position. It is critical to consider that these peak values can differ significantly with posture, which can differ between the CPET and hypogravity analogue (e.g., horizontal/HUT suspension). For example, maximal oxygen uptake can be reduced by ∼20% in a supine posture (compared to upright) during graded exercise testing to volitional exhaustion on a cycle ergometer due to an ∼10% decline in both peak heart rate and stroke volume (Dillon et al., 2021). A supine posture also lowers peak power output, work and gross efficiency, with the first and second ventilatory breakpoints occurring at lower intensities during cycling (Dillon et al., 2021; Wehrle et al., 2021). These postural effects can have substantial implications for interpreting energy expenditure, fatigue, lactate and ventilatory/gas exchange thresholds, thermoregulation and perceptual responses during movement in simulated hypogravity environments (Dillon et al., 2021; Fois et al., 2022; Whittle et al., 2022).

Human movement in hypogravity (e.g., for durations of minutes to hours) can be studied using bodyweight suspension analogues. However, it is important to recognise that these studies typically involve an immediate transition from 1 *g* to the target hypogravity level, during which only short‐term effects manifest. In contrast, during missions to the Moon and Mars, astronauts will undergo a range of physiological adaptations due to prolonged exposure to altered gravity environments during transit and after landing. Bodyweight suspension analogues, therefore, have value for investigating how human movement and the physiological responses to such movement may change following a period of disuse (e.g., bedrest, dry immersion) or spaceflight. This approach can provide mission‐specific insights by potentially revealing new phenomena that arise from disuse or spaceflight exposure or by identifying whether known responses to hypogravity become attenuated or exacerbated.

The biomechanical and physiological similarities in human movement between actual and simulated hypogravity within suspension analogues are not well understood. De Witt and colleagues identified that walking and running on a treadmill whilst loaded to ∼55% and 88% bodyweight during horizontal suspension compared well to actual microgravity facilitated by parabolic flight, with many kinetic and kinematic similarities (De Witt et al., 2010). Whilst some outcomes were relatively comparable between settings (e.g., ankle and knee range of motion), others, however, displayed large differences (De Witt et al., 2010). For example, hip flexion and maximum trunk angle were ∼15–25° and ∼10° greater and contact times and peak impact forces were ∼25–50% shorter and ∼25% lower, respectively, during parabolic flight compared to horizontal suspension (De Witt et al., 2010). Though this study examined locomotion in simulated microgravity when the body was loaded via a bungee system, it does raise important questions relating to how comparable human movement is between real and simulated hypogravity environments, which requires further comparative research. Factors such as dust, uneven terrains, pressurised spacesuits and altered vestibular inputs will be present on the Moon/Mars, which must also be considered (Lacquaniti et al., 2017).

### 4.3 Future directions

The expansion of humanity to distant terrestrial bodies warrants research into the effect of hypogravity on the human body, and whole‐body suspension analogues can play a pivotal role in accelerating our understanding in this area. The VGSS remains in ongoing development, where system upgrades will help refine and streamline the suspension process and improve the accuracy of simulating hypogravity. Plans are underway to reconstruct the VGSS in the North East Space Skills and Technology Centre (NESST) at Northumbria University in a laboratory that will allow the length of the suspension ropes to be increased significantly, thereby reducing the additional axial acceleration acting on the body during suspension due to the pendulum effect. This development will improve the fidelity of the simulation and broaden the scope of research that can be performed within the VGSS to inform upcoming missions to the Moon and Mars.

### 4.4 Conclusion

Hypogravity analogues provide valuable platforms from which to examine human biomechanical and physiological responses to reduced gravitational loading. Whole‐body suspension analogues can play a key role in accelerating human movement research in hypogravity environments; however, only a limited number of suspension analogues exist globally. This technical report introduced VGSS, a new micro‐ and hypo‐gravity suspension analogue, and demonstrated its ability to accurately simulate hypogravity in humans using HUT suspension with video footage showcasing locomotion and jumping movements performed in simulated lunar gravity.

## AUTHOR CONTRIBUTIONS

The study was conducted in the Aerospace Medicine & Rehabilitation Laboratory (Northumbria University, UK). All those who qualify for authorship are listed. All authors contributed to (1) the conception or design of the work, (2) the acquisition, analysis or interpretation of data for the work and (3) drafting the work or revising it critically for important intellectual content. All authors have read and approved the final version of this manuscript and agree to be accountable for all aspects of the work in ensuring that questions related to the accuracy or integrity of any part of the work are appropriately investigated and resolved. All persons designated as authors qualify for authorship, and all those who qualify for authorship are listed.

## CONFLICT OF INTEREST

None declared.

## FUNDING INFORMATION

None.

## Supporting information



Supporting Video 1 ‐ Jumping in Simulated Lunar Gravity.

Supporting Video 2 ‐ Locomotion in Simulated Lunar Gravity.

## Data Availability

All relevant data can be found within the article.
